# Impact of Praziquantel Mass Drug Administration on Schistosomiasis: A Comparison of Prevalence and Risk Factors Between Treated School Aged Children and Untreated Adults in Abuja, Nigeria

**DOI:** 10.3390/ijerph22050672

**Published:** 2025-04-24

**Authors:** Solomon M. Jacob, Sophie Y. Akinbo, Akinola S. Oluwole, Temitope Agbana, Zainab Omoruyi, Michael A. Okungbowa, Jan-Carel Diehl, Fredrick O. Akinbo

**Affiliations:** 1 Department of Medical Laboratory Science, University of Benin, P.M.B. 1154, Benin City 300283, Nigeria; owukpa2000@yahoo.com (S.M.J.); zainab.omoruyi@uniben.edu (Z.O.); michael.okungbowa@uniben.edu (M.A.O.); fredrick.akinbo@uniben.edu (F.O.A.); 2 Department of Public Health, Federal Ministry of Health, P.M.B. 083, Garki, Abuja 900108, Nigeria; 3 Department of Molecular Biology Diagnosis, Northwestern Memorial Hospital, Chicago, IL 60611, USA; sophieakinbo@gmail.com; 4 Sightsavers Country Office, Abuja 900271, Nigeria; aoluwole@sightsavers.org; 5 AiDx Medical Bv, 2641 KM Pijnacker, The Netherlands; t.e.agbana@tudelft.nl; 6 Sustainable Design Engineering, Delft University of Technology, 2628 CE Delft, The Netherlands

**Keywords:** schistosomiasis, endemicity, school-aged children, praziquantel effect, Abuja

## Abstract

**Introduction:** One of the global strategies for the elimination of schistosomiasis is by Mass Drug Administration (MDA) of a single oral dose of praziquantel (40 mg/kg) without a prior individual diagnosis, with a target of >75% treatment coverage among school-aged children. This study was conducted to determine the endemicity of schistosomiasis among school-aged children and adults in Abuja, Nigeria. **Methods:** A total of 1370 participants were recruited, which consisted of 667 (48.67%) males and 703 (51.31%) females. Urine and stool specimens were collected from each participant and analyzed using standard procedures. **Results:** The overall prevalence of schistosomiasis was 27.5% in this study with Abuja Municipal having the highest prevalence of 49%, while the least (6.1%) was reported in Bwari LAC. The prevalence of schistosomiasis significantly differs (*p* < 0.05) between the area councils. The location of communities significantly affected the prevalence of schistosomiasis in Abaji, AMAC, and Gwagwalada LACs (*p* < 0.005). The *Schistosoma* recovered in this study were *S. haematobium* and *S. mansoni*. The prevalence of schistosomiasis increased from the baseline of 21.1% to 49% in Gwagwalada LAC. Gender significantly affected the prevalence of schistosomiasis as more males were infected (33.1%) than their female counterparts (22.2%) (*p* < 0.05). The prevalence of schistosomiasis was 31% and 23.9% among SAC and adults, respectively. The participants’ activities in the river significantly affected the prevalence of schistosomiasis in this study (*p* < 0.05). **Conclusions:** The clamour for urgent government and non-government intervention through alternate sources of water like boreholes or pipe-borne water, as well as implementing a behavioural change campaign across the communities to prevent the recurrence, are advocated.

## 1. Introduction

Human schistosomiasis is an acute and chronic parasitic disease caused by infection with digenetic trematodes of the genus *Schistosoma* [1]. It is a major public health problem affecting approximately 240 million people worldwide with 90% of cases in sub-Saharan Africa, and causes approximately 70 million disability-adjusted life years lost [2,3]. According to a WHO report from 2017, an estimated 700 million people are at risk of infection, and approximately 200,000 annual deaths are from schistosomiasis alone [4]. Schistosomiasis is ranked second only to malaria in terms of socioeconomic impacts. It is a major source of morbidity and mortality for developing countries in Africa, South America, the Caribbean, the Middle East, and Asia [5]. According to the literature, an infection with schistosomiasis occurs when an individual is exposed to the contaminated water during routine agricultural, domestic, occupational (sand harvesting, fishing), and recreational activities. Children of school age are particularly vulnerable to chronic infections that can impair mental and physical development and reduce school attendance and educational achievement [6]. The global strategy for schistosomiasis control/elimination is the mass treatment of school-age children (SAC) with a single oral dose of praziquantel (40 mg/kg) without a prior individual diagnosis at >75% treatment coverage [7]. This is in addition to other measures such as snail control, and water, sanitation and hygiene (WASH) towards the attainment of the 2030 elimination goal as stipulated by WHO [3]. Recognizing the centrality of school-age children (SAC) to the response to helminth infections, in 2001, the 54th World Health Assembly passed a resolution to provide regular deworming treatment to 75 percent of school-age children at risk [8]. This forms the bedrock of current schistosomiasis control with praziquantel targeted towards school-age children. Prevalence for schistosomiasis is classified as Low (1–<10%), Moderate (10–<50%), and High (>50%), with all classifications previously recommended to receive MDA annually. This strategy has now been refined such that moderate and high endemicity now receive MDA annually while low endemicity is recommended for a test, and treatment strategy [9]. Nigeria is the most endemic country with schistosomiasis in Africa, with an estimated 134,073,166 people living in endemic communities at risk of the infection and requiring preventive chemotherapy [10]. Schistosomiasis is endemic in all the 36 states of Nigeria including the federal capital territory, Abuja.

## 2. Problem Statement

A baseline mapping survey was conducted in Nigeria between 2010 and 2014 to establish the prevalence of Schistosomiasis [11,12]. LACs (the administrative units for treatment decisions) were stratified to receive MDA for the disease either annually or every other year. In Abuja, six (6) Local Area councils (LACs) were identified as endemic and have been receiving praziquantel for mass distribution among school-age children who were the target of the WHO praziquantel donation program. The treatment program excluded other age groups, such as women of child-bearing age and other adults in Abaji, Abuja Municipal Area Council, Kwali, Kuje, Bwari, and Gwagwalada LACs. Children in Gwagwalada and Kwali LACs received treatment in 2014 but recorded low therapeutic coverages of 4.9% and 9.3%, respectively. Treatment was extended to the other LACs in 2015 but retracted in 2016, 2017, and 2018. Full treatment was re-established across all the LACs from 2019 to 2022, with almost all the LACs achieving about 100% therapeutic coverage [13]. Despite this treatment record spanning almost a decade, outbreak of the disease is periodically reported across several communities in Abuja by personal communications from community members and the FCT neglected Tropical Diseases (NTD) control program. This study aimed at determining the impact of mass distribution of praziquantel for the treatment of schistosomiasis among school-age children and compare the current prevalence and risk factors among this age group with others who were excluded from the treatment program in four (4) Area Councils of Abuja, Nigeria.

## 3. Materials and Methods

### 3.1. Study Area

**Figure 1:**
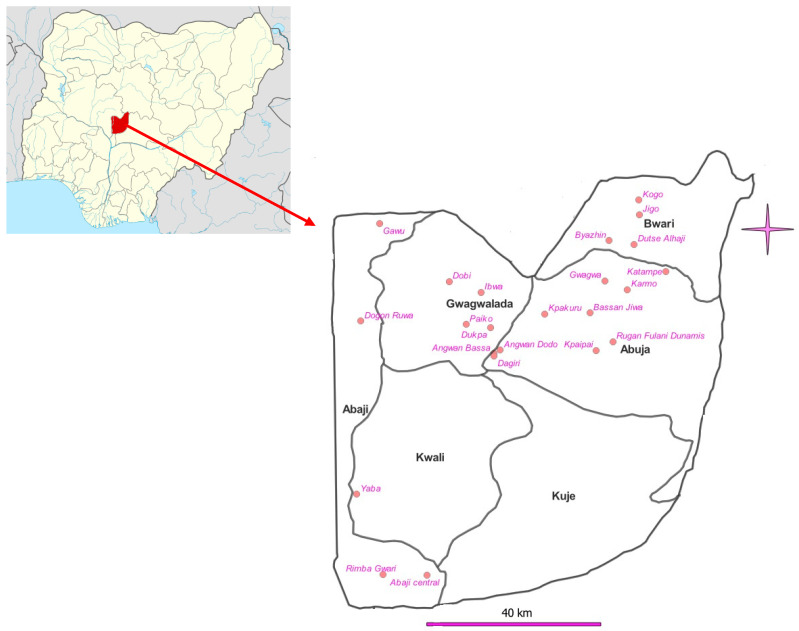
Map of Nigeria showing the study area.

The study was conducted in 4 Local Area Councils (LACs) of Abuja, Nigeria (Figure 1). The selected communities include Abaji Central, Dogon Ruwa, Gawu, Rimba gwarri and Yaba in Abaji Area Council; Bassan Jiwa, Gwagwa, Karmo, Kpaipai, Rugan Fulani Dunamis and Toge Sabo in Abuja Municipal Area Council; Byazhin, Dutse Alhaji, Jigo, Katampe, Kogo, Shere, War college Camp and Ushafa in Bwari Area Council; Angwan Bassa, Angwan Dodo, Dagiri, Dobi, Dukpa, Ibwa, Kpakuru, Kpakuru Sarki and Paiko in Gwagwalada Area Council. All the LACs have reported having between 3 and 5 annual rounds of praziquantel treatment. These communities were purposively selected as the baseline sites in the 2014 studies. However, 3 communities each were added in Gwagwalada and Bwari LACs based on reports of outbreak by community members

The geographical coordinate of the study area lies between latitude 8.25 and 9.20 N of the equator and longitude 6.45 and 7.39 E of the Greenwich meridian. It is situated within the savannah region with moderate climatic conditions. The high altitudes of the rolling terrain of the FCT act as a moderating influence on the weather of the territory. Abuja has a population size of 4,026,000 as of 2024, as projected from the 2006 population census [13]. The primary economic activity in the Federal Capital Territory (FCT) is agriculture, which produces crops like yam, millet, corn, sorghum, and beans. The majority of the population are dairy farmers from the Gwari, Koro, Ganagana, Gwandara, Afo, and Bassa ethnic groups. Hausa and Fulani also live in the territory. While others engage in trading, the city center boasts a sizable number of civil servants who service the seat of governance. Rainfall reflects the territory’s location on the windward side of the Jos plateau and the zone of rising air masses. The annual total rainfall is between 1100 and 1600 mm, with wet and dry seasons. Several freshwater habitats intersect the study area, some of which include ponds, streams, dams, and tributaries of the Gurara River stretching from Kaduna State. These water bodies form the major source of water supply to the residents of the study area. During dry seasons, activities increase around these water bodies as people converge to use them for domestic, agricultural, and recreational activities, all of which could predispose them to schistosomiasis.

### 3.2. Study Design

The study was cross-sectional, targeting school-age children in schools and adults in the communities. The identification of villages was aided by data from the National Schistosomiasis Elimination Program. Village selection considered the baseline sites from the 2014 study and personal communication of history of outbreak of the disease. as local public health officials provided lists of potential schools and villages for inclusion.

A range of 50–55 subjects of both sexes was sampled from each selected community, whereas in each village across the 4 LACs (Abaji, AMAC, Bwari, and Gwagwalada), a total of ±25 school-age children (SAC) were selected in the school using a systematic random sampling frame, ±25 adults were also selected from 25 random households at 1 participant per household from the community. The ages of the participants ranged from 5–14 years for SAC and 15 years and above for the adult population. Enrolled participants were provided with instructions on how to collect the specimen after completing the questionnaire and the consent forms.

The sample size was calculated using the Cochran formula described by Bartlett et al. [14].

n_o_ = Z^2^pq

e^2^

where: e is the desired level of precision at 95% confidence (i.e., the margin of error)

p is the baseline prevalence (estimated proportion of the population that has the attribute in question)

q is 1 − p, while the Z value at 95% confidence level is 1.96.

Assuming a baseline prevalence of 25.1%, 21.12%, 7.48%, 51.9%, 20.4%, and 27.89% for Abaji, AMAC, Bwari, and Gwagwalada, respectively, the expected sample sizes, including 10% attrition rates, the sample size calculated were:

Abaji LGA: n_o_ = (1.96)2 × 0.251 × (1 − 0.251)/0.05^2^ + 10% = 318

AMAC LGA: n_o_ = (1.96)2 × 0.2112 × (1 − 0.2112)/0.05^2^ + 10% = 281

Bwari LGA: n_o_ = (1.96)2 × 0.0748 × (1 − 0.0748)/0.05^2^ + 10% = 117

Gwagwalada LGA: −n_o_ = (1.96)^2^ × 0.519 × (1 − 0.519)/0.05^2^ + 10% = 423

Following WHO guideline of 50–55 participants per community [15], ~25 SAC and 25 Adults within household were targeted in each selected community from the 4 LACs

### 3.3. Specimen Collection

Samples for this study were collected between January and May 2023. Each selected participant was provided with a capped sterile specimen bottle and instructed to fill it with about 20 mL of clean catch, midstream urine samples. participants were subjected to a little exercise to agitate their bladder and carefully instructed with illustration aid. A second capped sterile specimen bottle with an applicator stick was also provided along with tissue paper to clean up after defecation. They were instructed to pass the stool on a clean white paper and use the applicator stick to transfer about 5 g of the stool into the specimen bottle provided. The two (2) capped specimen bottles were labeled with unique identification (ID) numbers. Samples were collected between 10.00 am and 2.00 pm and transported to the laboratory not later than two hours after collection. All samples were analyzed on the same day. The study also assessed exposure to praziquantel and other facilitating factors that aid the transmission of schistosomiasis in these local area councils. The urine and stool specimens were processed using standard methods.

### 3.4. Urine Filtration Technique

In the laboratory, each urine sample was homogenized by gentle agitation and 10 mL was filtered using urine filtration technique to concentrate eggs of schistosome on membrane filter 13 mm and 12 micrometer pores (Starlitech) as described by Lengeler et al. [16]. Intensity of *S, heamatobium* was reported as the number of ova/10 mL of urine, and were categorized as light infection (˂50 ova/10 mL of urine) or heavy infection (>50 ova/10 mL of urine).

### 3.5. Kato-Katz Technique for Stool Examination

Stool samples collected from the schools and communities were also examined for parasite eggs using the Kato-Katz technique described by WHO [17]. Intensity of *S. mansoni* infection was reported as the number of eggs per grams (epg) of stool and were categorized as light infection (1–99 epg), moderate infection (100–399 epg) or heavy infection (≥400 epg) based on WHO guidelines

### 3.6. Statistical Analysis

Data was entered into Microsoft Excel and imported into IBM SPSS version 20 which was used for statistical analysis. Descriptive statistics was used to describe the occurrence of infection and results were considered significant where *p* < 0.05. The Pearson Chi-square test association between schistosomiasis infection status and other variables like sex, age, and exposure to risk factors. Pearson’s Chi-square was also used to compare the proportion of infections between age categories, communities, and area councils. Mann-Whitney U test was used to compare the intensity of infection between the SAC population and adults for age.

## 4. Results

### 4.1. Demographic Characteristics of the Studied Population

Table 1 below shows demographic characteristics of the participants. A total 667 (48.67%) of the respondents were male and 703 (51.31%) were female. Generally, there were more male participants than female participants in all the LACs except in Abaji area council where there were more female participated in the study than male. The sources of water used by study participants vary from one area council to the other. 287 (20.9%) participants uses both well and rain water, 300 (21.9%) uses borehole, 238 (17.4%) use tap water, 324 (23.6%) uses the river only, 93 (6.8%) uses well, rain and river, 78 (5.7%) uses well, rain, bore hole and river while 50 (3.6%) uses borehole and river as source of water. Participants that use the river as a source of water, carried out different activities in the river. These activities include Fetching water, Swimming, Bathing, Washing, crossing the river and fishing. The most activities carried out was swimming 159 (29.2%) and the least activity carried out was Fishing.

### 4.2. Prevalence of Schistosomiasis by Local Area Councils

The overall prevalence of schistosomiasis, a cumulative of *Schistosoma haematobium* and *Schistosoma mansoni* was 27.5% in this study. The aggregate prevalence of schistosomiasis by LACs shows that AMAC had the highest prevalence of 49% while the least was recorded in Bwari Area Council with a prevalence of 6.1%. The Prevalence of schistosomiasis significantly differs (*p* < 0.05) between the area councils. Based on WHO classification of prevalence, three of the council areas (Abaji, AMAC, and Gwagwadala) fell within the moderate prevalence while Bwari fell within the low prevalence category (Table 2).

Out of the 27 communities studied, four communities fell within the high prevalence category (>50%). The location of communities significantly affected the prevalence of schistosomiasis in Abaji, AMAC and Gwagwalada LACs (*p* < 0.005). This includes Abaji central from Abaji Council Area, Bassa Jiwa and Gwagwa from AMAC, and Angwan Bassa from Gwagwalada Council Area. Fourteen (14) communities were within the moderate prevalence category (>10 and <50%) namely two communities from Abaji LACs (Dogon Ruwa and Yaba), four communities from AMAC LACs (Karmo, Kpaipai, Rugan Fulani Dunamis and Toge Sabo) one community from Bwari LCA (Byazhin) and seven communities from Gwagwalada LACs (Angwan Dodo, Dagiri, Dobi, Ibwa, Kpakuru, Kpakuru Sarki, Paiko). Seven communities were within the low prevalence category (<10%), four of which are from Bwari LACs (Dutse Alhaji, Jigo, Katampe and Shere), two from Abaji LACs (Gawu and Rimba gwari) and one from Gwagwalada LACs (Dukpa). However, there was no case of schistosomiasis was found in Kogo community in Bwari LACs (Table 3).

### 4.3. Prevalence and Intensity by Schistosoma Species

**Figure 2:**
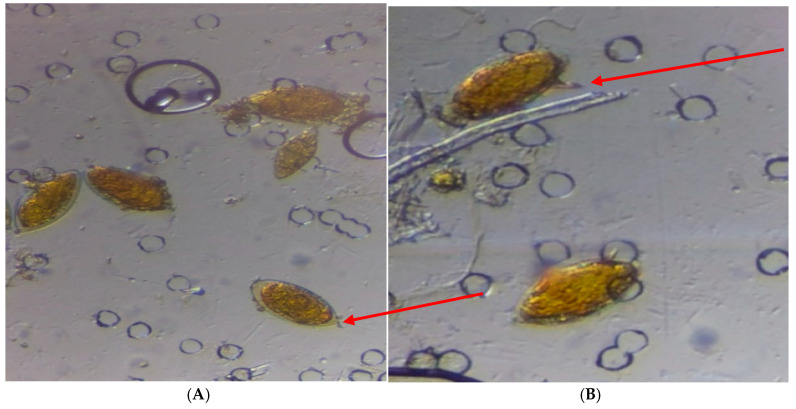
Ova of *S. haematobium* (**A**) with terminal spine and Ova of *S. mansoni* (**B**) with lateral spine.

Given the spine’s position, two species of schistosomes were prevalent in the study area (Figure 2). These were urogenital *S. haematobium* with terminal spine and intestinal *S. mansoni* with lateral spine. Prevalence of each of the two species vary from one LAC to the other. For *Schistosoma haematobium* species, highest prevalence of 45.1% was observed in AMAC, followed by Gwagwalada (22.4%) and the least (3.1%) was observed in Bwari Local Area Council (Table 3). In contrast, the prevalence of *S. mansoni* species was higher in Abaji LAC (14.6%), followed by Gwagwalada LAC (11.5%) and the least in Bwari LAC (3.4%) (Table 4). Analysis of intensity of infection within each council shows that 33.3% and 17.5% of subjects infected with *S. haematobium* from AMAC and Gwagwalada had heavy intensity of infection respectively while less than 10% of participants infected with *S. haematobium* from both Abaji, and Bwari had heavy infection. Similarly, participants infected with *S. mansoni* from AMAC had higher percentage of heavy intensity when compared to other LCAs that showed moderate intensity of infection (Table 4 and Table 5).

### 4.4. Comparison of the Impact Prevalence with Baseline Prevalence

**Figure 3:**
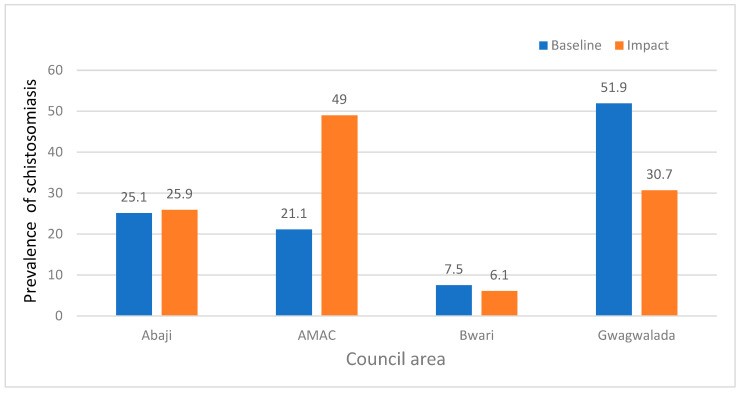
Comparison of the impact of Praziquantel MDA in 2014 with the report of 2022.

Comparing the prevalence at baseline with that observed in this study, we observe a significant reduction in the prevalence of schistosomiasis from 51.9% to 30.7% in Gwagwalada LAC. There was also a slight reduction in the prevalence (7.5% to 6.1%) in Bwari LAC. However, there was a significant increase in the prevalence of schistosomiasis in AMAC from 21.1% at baseline to 49%, and from 25.1% to 25.9% in Abaji area council (Figure 3).

### 4.5. Relationship Between Gender and Schistosoma Infection

Generally, gender significantly affected the prevalence of schistosomiasis with the male participants having the highest prevalence (33.1%) when compared with their female counterparts (22.2%) (*p* < 0.05). In addition, male and female participants from AMAC had the highest prevalence of schistosomiasis (51.9%, and 45.9%, respectively) when compared with the other (Table 6).

### 4.6. Relationship Between Schistosomiasis Infection and Age Category (SAC and Adult)

Age of participants significantly affected the prevalence of schistosomiasis in this study (*p* < 0.05). An overall prevalence of 31% observed among SAC and 23.9% among adult participants. All the area councils recorded similar patterns of prevalence among SAC and Adults with the SAC showing the highest prevalence (Table 7).

Age did not influence the intensity of infection with *S. haematobium* species as the prevalence of light and heavy-intensity infection between the age categories was not significantly different in all the local area councils (*p* > 0.05) (Table 8). Similarly, the age categories of participants did not significantly affect the intensity of *S. mansoni* in all the LACs (*p* > 0.05). The majority of the communities within Abaji LAC showed light intensity whereas the Bwari, Gwagwalada, and AMAC LACs were observed to have a moderate intensity of *S. mansoni* infection among the SAC category. A similar trend was observed in the communities within AMAC and Gwagwalada LACs showed moderate intensity of *S. mansoni* infection among the adult category. Meanwhile, the adult participants in Abaji and Bwari LCAs showed the light intensity of *S. mansoni* infection (Table 9).

### 4.7. Association Between Sources/Activities in the River and Prevalence of Schistosomiasis

Participants who used river water alone or in combination with other water sources had *Schistosoma* infection in this study. However, there was no case of *Schistosoma* infection among those who used well and rain, borehole, and tap water (Table 10).

Activities like swimming and fishing show a higher prevalence of schistosomiasis (83% and 90.9%, respectively), depicting a strong association between frequent visit to the water bodies during these activities and the risk of acquiring schistosomiasis infection. In contrast, we observed that crossing water showed a lower prevalence of the parasites (55.4%). In addition, the activities of participants in the river significantly affected the prevalence of schistosomiasis in this study (*p* < 0.05) (Table 11).

## 5. Discussion

Schistosomiasis is one of the neglected tropical diseases targeted for elimination by 2030 according to the WHO Roadmap 2030 [9]. Consequently, each endemic country is working to meet this target by reviewing its strategies for control. Nigeria has been observed to have the highest number of schistosomiasis cases in the world—in the African region, over 26% of people requiring chemotherapy reside in Nigeria [15]. Significant gaps in epidemiological data create difficulties in understanding the true distribution of the disease and necessary intervention [15]. particularly, in Nigeria. A recommended approach to understanding the progress made in the fight against the disease is conducting an impact assessment study after rounds of effective treatment [18].

The prevalence of schistosomiasis observed in our study area reveals significant disparities in the different area councils particularly; the Abuja Municipal Area Council (AMAC) which recorded the highest prevalence of 49%, indicating a considerable public health concern in this LAC. In addition, the Abuja Municipal Council recorded a prevalence (49%) that doubles the baseline report of 21.1% in 2014 despite the praziquantel mass treatment. This finding is consistent with the studies of [19,20]. where schistosomiasis transmission persisted despite several rounds of MDA. In contrast, Bwari Area Council showed the least prevalence of 6.1%. This difference in the prevalence of schistosomiasis among the local area councils in the FCT highlights the heterogeneous distribution of the infection across the study area, which is relevant for developing targeted intervention strategies in combating the parasitic disease. The overall prevalence of 27.5% observed in the FCT is reflective of a moderate endemicity category that necessitates immediate public health surveillance and interventions. The prevalence reported in this study is higher than the 4% observed by Oluwole et al. [21]. in Ogun State but lower than the 51.2% observed among primary school children at Yewa North LGA in Ogun State by Alabi et al. [22]. Application of WHO classification provides a framework for guiding decision-making [23]. to ensure that resources are shared appropriately to manage and mitigate *Schistosoma* infection in all the LACs of the FCT. Based on WHO guidelines on the classification of the prevalence of schistosomiasis, Abaji, AMAC, and Gwagwalada LACs have been classified as moderate prevalence. This suggests that regular MDA and implementing community awareness initiatives would go a long way in mitigating disease transmission. However, Bwari LAC falls into the low prevalence category; the infection in this LAC should not be overlooked in public health planning.

This study identified fourteen (14) communities classified under moderate prevalence (>10 and <50%). This category includes Dogon Ruwa and Yaba, from Abaji LAC; Karmo, Kpaipai, Rugan Fulani Dunamis, and Toge Sabo from AMAC; Byazhin from Bwari LAC and Angwan Dodo, Dagiri, Dobi, Ibwa, Kpakuru, Kpakuru Sarki, and Paiko from Gwagwalada LAC. The moderate prevalence observed in these communities reflects a substantial risk of infection that may necessitate targeted interventions like regular health education and MDA to reduce disease incidence in the LACs. Conversely, the following communities namely Dutse Alhaji, Jigo, Katampe, and Shere in Bwari LAC; Gawu and Rimba Gwuri from Abaji LAC; and Dukpa from Gwagwalada LAC were grouped as low prevalence category (<10%). Surprisingly, no case of schistosomiasis was recorded in Kogo community, suggesting the potential effectiveness of existing public health measures or environmental factors that may be responsible for limiting transmission of the parasite. The inconsistency in prevalence among the communities underscored the need for targeted public health responses involving the geographical, social, and environmental determinants of schistosomiasis transmission [24,25]. For all the communities in the FCT, it is imperative to implement increased frequency of praziquantel treatment, community initiatives to educate the people about the risks and transmission pathways of the disease as well as improvements in water sanitation facilities to reduce contact with contaminated water bodies.

In this study, two species of schistosomes were recovered namely the urogenital *Schistosoma haematobium*, the intestinal *Schistosoma mansoni*. The prevalence of these species showed notable variation across the FCT. *Schistosoma haematobium* demonstrated the highest prevalence of 45.1% in the Abuja Municipal Area Council (AMAC), followed by 22.4% in Gwagwalada, while Bwari had the least prevalence at 3.1%. However, *S. mansoni* was primarily found in Abaji LAC (14.6%), followed closely by Gwagwalada LAC at 11.5%, and again Bwari LAC reported the least prevalence at 3.4%. The difference in prevalence in the LACs highlights the localized nature of schistosomiasis transmission that may be attributed to environmental, socio-economic, and behavioral factors commonly found in each council area.

The AMAC not only had the highest prevalence of *S. haematobium* (29.5%) but also recorded a greater percentage of heavy-intensity infections (33.3%), defined by >50 eggs per 10 mL of urine. Comparatively, only 17.5% of those from Gwagwalada had heavy intensity, whereas <10% of Abaji and Bwari participants showed similar intense infection categories. This may indicate that AMAC may be a focal point for severe cases of schistosomiasis, necessitating targeted public health interventions to address the significant burden of disease in that locality. Similarly, it was observed that AMAC also had a higher percentage of respondents with heavy-intensity infection of *S. mansoni* when compared to other LACs. This finding suggests that while *S. mansoni* may be less prevalent in this study, it still poses a significant health risk in certain areas, particularly in AMAC. *S. mansoni* infections usually present with symptoms related to intestinal issues, which can complicate public health responses since they may be confused with other gastrointestinal conditions [26]. As public health strategies evolve, a deeper understanding of the environmental and socio-behavioral factors contributing to schistosomiasis transmission is essential which can guide effective resource allocation and intervention designs, ensuring that control measures not only address current prevalence but also incorporate sustainable practices to reduce future risks [27].

There was a significant reduction from 51.9% at baseline to 30.7% in the prevalence of schistosomiasis in Gwagwalada LAC. This reduction is evidence of potential successes in control measures implemented in this area. Conversely, the Abuja Municipal Area Council had a significant increase in schistosomiasis prevalence from 21.1% at baseline to 49% in this study. The spike in the prevalence of schistosomiasis could signal emerging risks and necessitates a consideration of new environmental or sociopolitical factors potentially influencing transmission dynamics. Meanwhile, the prevalence of schistosomiasis in Abaji remained stable when compared with the baseline report (25.1% and 25.9%), suggesting that the control efforts have managed to maintain a non-fluctuation status.

Generally, male participants had a greater prevalence of 33.1% compared to 22.2% for females, indicating that gender significantly affected the prevalence of schistosomiasis (*p* < 0.05). This finding may be attributed to differences in risk exposure levels between genders where males are more likely to engage in outdoor activities such as fishing and bathing in contaminated water bodies, where they are more vulnerable to infection. Male AMAC participants had the highest prevalence of 51.9%, followed by female participants (45.9%). The finding of more males been infected with schistosomiasis is consistent with the reports of Woldeyohannes et al. [28]. and Balogun et al. [29]. among school-aged children in Ethiopia and Jigawa State in Nigeria. The reason for this finding could be that the male participants had a higher frequency of contact with contaminated water bodies than their female counterparts while assisting with family outdoor chores like herding cattle, fishing, and farming. In addition, male participants could be more engaging in outdoor plays and recreational activities than females, which may predispose the boys to higher risks of schistosomiasis. This finding highlights the necessity of gender-specific initiatives that raise awareness among vulnerable groups. Programs promoting community-wide sanitation improvements can significantly reduce or eliminate infection rates and improve public health.

The age of participants significantly affected the prevalence of schistosomiasis in this study with the school-aged children (SAC) who benefited from the annual MDA having a higher prevalence of schistosomiasis (31%) than that of the adults that were not treated (23.9%), justifying why the schistosomiasis control programme prioritize treatment of school aged children [9]. This is in tandem with a previous study that identifies school-aged children as a high-risk group due to increased exposure to contaminated water bodies during recreational activities, agricultural labor, and domestic chores [27].

Studies have shown that children often engage in water contact activities such as swimming, fishing, and washing, increasing their risk of encountering the parasite [22]. The mean intensity of *Schistosoma haematobium* infection in the school-aged children (SAC) population is significantly higher than that observed in adults (*p* < 0.05). This suggests that school-aged children are at higher risk of acquiring schistosomiasis and tend to have higher levels of infection severity compared to adults. The higher intensity of infection in younger population could be attributed to several factors, including increased exposure to contaminated water sources, which are common in the daily activities of school-aged children.

Despite recommendations by the WHO in 2010 to treat 2- and 3-year-old preschool-aged children (PSAC) with praziquantel in off-license settings, [15]. guidelines are difficult to implement. Praziquantel is only formally licensed for 4-year-olds and above, and the global community continues to discuss the best tools and approaches for targeting the PSAC age group. From a mathematical point of view, treating the SAC population alone, which represents only about 20–26% of the population, cannot lead to elimination of the disease, considering that the SAC population will return to the community where 80% of the population are not treated and become reinfected by them. In addition, the recent global attention on the issue of female genital schistosomiasis (FGS), a complication from schistosomiasis infection without treatment, although it can be prevented by treatment with praziquantel [30]. This reason has further justified the need to scale up treatment for everyone at risk of schistosomiasis infection. It is against this backdrop that the WHO released a new guideline in 2022 encouraging all endemic communities to include all at-risk populations starting from age 2 years upward, including pregnant women after the first trimester and lactating mothers in MDA for schistosomiasis control [9]. Notably, all local area councils in this study showed similar patterns of prevalence of schistosomiasis among school-aged children and adults. This consistency suggests underlying common factors across the councils contributing to schistosomiasis transmission. Looking at the objective of the Leave No One Behind Goal, a schistosomiasis control program that does not include the treatment of other at-risk groups is denying the individual the right to good health [31].

This study observed that the age categories of participants did not significantly affect the intensity of *Schistosoma mansoni* infection across all local council areas (*p* < 0.05). This suggests that regardless of whether participants are school-aged children (SAC) or adults, the level of infection intensity remains relatively consistent across different age groups. However, infection intensity varies by the community as Abaji LAC had predominantly light-intensity infections of *S. mansoni*, but Bwari, Gwagwalada, and Abuja Municipal Area Councils had moderate-intensity infections among SAC. This observed disparity suggests that localized factors influence schistosomiasis transmission dynamics, which could be connected to water contact patterns or environmental conditions in these councils. Within the AMAC and Gwagwalada LACs, moderate intensity of schistosomiasis was observed in the adult category, indicating that adults in these areas experience a similar burden as SAC. Conversely, adults in Abaji and Bwari LCAs predominantly demonstrated the light intensity of infection. This finding highlights the complex interplay between community characteristics, water exposure patterns, and schistosoma infection intensity across different age demographics.

Participants who relied on river water either exclusively or in combination with other sources had *Schistosoma* infection. This finding observed that source of water significantly affected the prevalence of schistosomiasis infection in this study (*p* < 0.0001). Conversely, no infections were reported among participants that utilized well water, rainwater, boreholes, or tap water. This stark contrast emphasizes the heightened risk of schistosomiasis associated with using untreated surface water that harbors the parasite’s infective stage due to the presence of intermediate snail hosts.

Swimming and fishing were associated with high prevalence of 83% and 90.9%, respectively. This observation suggests a significant relationship between frequent and prolonged exposure to the river while swimming and fishing and the possibility of being infected by the parasite. On the contrary, less exposure risk was observed among participants that crossed the rivers (55.4%). This finding suggests public health interventions that target specific high-risk activities like recreational and occupational water use practices. Non-availability or lack of access to potable water increases the risk of schistosomiasis infections as the inhabitants of such communities are forced to go to the river, the source of infection since there is no alternative [32]. Hence, our findings corroborate previous evidence that reported high chances of re-infection of schistosomiasis in communities under MDA but lack access to potable water [20]. This finding, therefore, adds to the body of evidence supporting the fact that MDA alone cannot bring about the elimination of schistosomiasis without the successful implementation of water, sanitation, and hygiene (WASH) practices and behavioural change intervention [33,34,35]. Evidence from this study seems to re-emphasize the need for and importance of providing portable water in schistosomiasis endemic communities to reduce continuous exposure to sources of infection and break the schistosomiasis transmission cycle [36]. This was observed in some communities in our study as we observed that there were no cases of schistosomiasis infection among participants who had access to potable water like tap water, and boreholes. It is important to note that the provision of potable water in schistosomiasis endemic communities without community engagement and implementation of behaviour change components may result in the community not using the portable water provided for them [37].

## 6. Limitations of the Study

This study was unable to establish potential emergence of drug-resistant strains of *Schistosoma*, which might reduce the efficacy of MDA due to repeated use of praziquantel.

## 7. Conclusions

The overall prevalence of schistosomiasis was 27.5% in this study. AMAC had the highest prevalence of 49% of schistosomiasis while the least (6.1%) was reported in Bwari LCA. The prevalence of schistosomiasis significantly differs between the area councils (*p* < 0.05). The location of communities significantly affected the prevalence of schistosomiasis in Abaji, AMAC, and Gwagwalada LCAs (*p* < 0.005). *S. haematobium* and *S. mansoni* were the species observed among SAC and adult participants in this study. The prevalence of schistosomiasis increased from the baseline of 21.1% to 49% in Gwagwalada LCA. Gender significantly affected the prevalence of schistosomiasis as more males were infected (33.1%) than their female counterparts (22.2%) (*p* < 0.05). The prevalence of schistosomiasis was 31% and 23.9% among SAC and adults, respectively. The participants’ activities in the river significantly affected the prevalence of schistosomiasis in this study (*p* < 0.05).

## 8. Recommendation

The clamor for urgent government and non-government intervention through alternate sources of water like boreholes or pipe-borne water, as well as implementing a behavioral change campaign across the communities to prevent the recurrence, are advocated.

## Figures and Tables

**Table 1:** Demographic characteristics of the studied population.

	Abaji n (%)	AMAC n (%)	BWARI n (%)	Gwagwalada n (%)	Total
**Sex**					
Male	121 (48.99%)	158 (51.63)	148 (41.34)	240 (52.29)	667 (48.67)
Female	126 (51.01%)	148 (48.37)	210 (58.66)	219 (47.71)	703 (51.31)
Total	247 (100%)	306 (100)	358 (100)	459 (100)	1370 (100)
**Age**					
SAC 5–15 years	123 (49.8%)	149 (48.7)	180 (50.3)	245 (53.4)	697 (50.9)
Adult > 15 years	124 (50.2%)	157 (51.3)	178 (49.7)	214 (46.6)	673 (49.1)
Total	247 (100%)	306 (100)	358 (100)	459 (100)	247 (100)
**Source of water** **for domestic use**					
Well/Rain	89 (36.0%)	25 (8.2%)	0 (0.0%)	173 (37.7%)	287 (20.9%)
Borehole	18 (7.3%)	73 (23.9%)	98 (27.4%)	111 (24.2%)	300 (21.9%)
Tap water	0 (0%)	0 (0%)	238 (66.5%)	0 (0%)	238 (17.4%)
River	113 (45.7%)	67 (21.9%)	15 (4.2%)	129 (28.1%)	324 (23.6%)
Well/Rain/River	8 (3.2%)	69 (22.5%)	3 (0.8%)	13 (2.8%)	93 (6.8%)
Well/Rain/Borehole/River	15 (6.1%)	43 (14.1%)	0 (0%)	20 (4.4%)	78 (5.7%)
Borehole and Rivers	4 (1.6%)	29 (9.5%)	4 (1.1%)	13 (2.8%)	50 (3.6%)
Total	247 (100%)	306 (100%)	358 (100%)	459 (100%)	1370 (100%)
**Activities in the river**					
Fetching water	27 (19.3%)	10 (4.8%)	1 (4.5%)	8 (4.6%)	46 (8.4%)
Swimming	44 (31.4%)	37 (17.8%)	21 (95.5%)	57 (32.6%)	159 (29.2%)
Bathing	15 (10.7%)	40 (19.2%)	-	39 (22.3%)	94 (17.2%)
Washing	30 (21.4%)	68 (32.7%)	-	43 (24.6%)	141 (25.9%)
Crossing river	24 (7.1%)	41 (19.7%)	-	18 (10.3%)	83 (15.2%)
Fishing	-	12 (5.8%)	-	10 (5.7%)	22 (4.0%)
Total	140 (100%)	208 (100%)	22 (100%)	175 (100%)	545 (100%)

**Table 2:** Prevalence of Schistosomiasis by Local Area Councils.

LAC	No. Tested	No. Positive (%)	*p*-Value	WHO Prevalence Categories
Abaji	247	64 (25.9)	<0.0001	Moderate
AMAC	306	150 (49)		Moderate
Bwari	358	22 (6.1)		Low
Gwagwalada	459	141 (30.7)		Moderate

**Table 3:** Prevalence of schistosomiasis by community.

Community	No. Tested	No. Positive	Prevalence of Schistosomiasis (%)	*p*-Value	Prevalence Level
**Abaji LCA**					
Abaji Central	48	24	50.0		High
Dogon Ruwa	51	22	43.1		Moderate
Gawu	50	4	8.0		Low
Rimba gwari	48	2	4.2		Low
Yaba	50	12	24.0		Moderate
**Total**	**247**	**64**	**25.9**	<0.0001 *	**Moderate**
**AMAC LCA**					
Bassan Jiwa	50	35	70.0		High
Gwagwa	50	44	88.0		High
Karmo	49	17	34.7		Moderate
Kpaipai	55	15	27.3		Moderate
Rugan Fulani Dunamis	50	23	46.0		Moderate
Toge Sabo	52	16	30.8		Moderate
**Total**	**306**	**150**	**49.0**	<0.0001 *	**Moderate**
**BWARI LCA**					
Byazhin	51	7	13.7		Moderate
Dutse Alhaji	50	4	8.0		Low
Jigo	54	3	5.6		Low
Katampe	52	5	9.6		Low
Kogo	50	0	0.0		Low
Shere	49	1	2.0		Low
War college Camp, Ushafa	52	2	3.8		Low
**Total**	**358**	**22**	**6.1**	0.0549	**Moderate**
**GWAGWALADA LCA**					
Angwan Bassa	50	36	72.0		High
Angwan Dodo	50	14	28.0		Moderate
Dagiri	54	22	40.7		Moderate
Dobi	50	16	32.0		Moderate
Dukpa	50	4	8.0		Low
Ibwa	50	7	14.0		Moderate
Kpakuru	51	14	27.5		Moderate
Kpakuru Sarki	50	6	12.0		Moderate
Paiko	54	22	40.7		Moderate
**Total**	**459**	**141**	**30.7**	0.00053 *	**Moderate**

* *p* < 0.05.

**Table 4:** Intensity of urinary schistosomiasis by local council areas.

LAC	No. Tested	No. Positive (%)	Light Infection (%)	Heavy Infection (%)	Total Egg Count	Mean Intensity	WHO Prevalence Categories
Abaji	247	33 (13.4)	30 (30.9)	3 (1.2)	621	19	LI
AMAC	306	138 (45.1)	92 (66.7)	46 (33.3)	14,838	108	HI
Bwari	358	11 (3.1)	10 (90.9)	1 (9.1)	212	19	LI
Gwagwalada	459	103 (22.4)	85 (82.5)	18 (17.5)	3237	31	LI

Key: LI = Light infection (<50 eggs/10 mL urine); HI = Heavy infection (>50 eggs/10 mL urine).

**Table 5:** Intensity of intestinal schistosomiasis by local council areas.

LAC	No. Tested	No. Positive (%)	LI (%)	MI (%)	HI (%)	Total Egg Count	Overall Mean Intensity	WHO Intensity Categories
Abaji	247	36 (14.6)	22 (61.1)	11 (30.6)	3 (8.3)	6024	167	MI
AMAC	306	33 (10.8)	6 (18.2)	20 (60.6)	7 (21.2)	8736	265	MI
Bwari	358	12 (3.4)	6 (50)	5 (41.7)	1 (8.3)	2304	192	MI
Gwagwalada	459	53 (11.5)	12 (22.6)	30 (56.6)	11 (20.8)	13,104	247	MI

Key: LI = Light infection (1–99 epg); MI = Moderate infection (100–399 epg); HI = Heavy infection (≥400 epg).

**Table 6:** Relationship between gender and *Schistosoma* infection.

LGA	Total Sampled	Male		Female	
		Sampled	Number Positive (Prevalence)	Sampled	Number Positive (Prevalence)
Abaji	247	121	37 (30.6%)	126	27 (21.4%)
AMAC	306	158	82 (51.9%)	148	68 (45.9%)
Bwari	358	148	12 (8.1%)	210	10 (4.8%)
Gwagwalada	459	240	90 (37.5%)	219	51 (23.3%)
Grand Total	1370	667	221 (33.1%)	703	156 (22.2%)

**Table 7:** Relationship between age category (SAC and Adult) and prevalence of schistosomiasis.

LAC	No. Examined	School-Aged Children		Adult	
		No. Tested	Number Positive (Prevalence)	No. Tested	Number Positive (Prevalence)
Abaji	247	123	38 (30.9%)	124	26 (21%)
AMAC	306	149	87 (58.4%)	157	63 (40.1%)
Bwari	358	180	12 (6.7%)	178	10 (5.6%)
Gwagwalada	459	245	79 (32.2%)	214	62 (29%)
Grand Total	1370	697	216 (31.0%)	673	161 (23.9%)

**Table 8:** Correlation between age category and intensity of *S. haematobium* infection.

LAC	No. Examined	School-Aged Children			Adult		
		No. Positive	Light Infection (<50 Eggs per mL)	Heavy Infection (>50 Eggs per mL)	No. Positive	Light Infection (<50 Eggs per mL)	Heavy Infection (>50 Eggs per mL)
Abaji	247	123	121 (98.4)	2 (1.6)	124	123 (99.2)	1 (0.8)
AMAC	306	149	244 (98.8)	3 (1.2)	157	133 (84.70)	24 (15.3)
Bwari	358	180	179 (99.4)	1 (0.6)	178	178 (100.0)	0 (0.0)
Gwagwalada	459	245	234 (95.5)	11 (4.5)	214	207 (96.7)	7 (3.3)
Grand Total	1370	697	661 (94.8)	36 (5.2)	673	641 (95.2)	32 (4.8)

**Table 9:** Correlation between age category and intensity of *S. mansoni* infection.

LAC	No. Examined	School-Aged Children				Adult			
		No. Positive	Light Infection (1–99 epg)	Moderate Infection (100–399 epg)	Heavy Infection (≥400 epg)	No. Positive	Light Infection (1–99 epg)	Moderate Infection (100–399 epg)	Heavy Infection (≥400 epg)
Abaji	247	21	14 (66.7%)	4 (19.0%)	3 (14.3%)	15	8 (53.3%)	7 (46.7%)	0 (0%)
AMAC	306	20	3 (15.0%)	13 (65.0%)	4 (20.0%)	13	3 (23.1%)	7 (53.8%)	3 (23.1%)
Bwari	358	7	3 (42.9%)	4 (57.1%)	0 (%)	5	3 (60.0%)	1 (20.0%)	1 (20.0%)
Gwagwalada	459	30	6 (20.0%)	16 (53.3%)	8 (26.7%)	33	6 (26.1%)	14 (60.9%)	3 (13.0%)
Grand Total	1370	78	26 (33.3%)	37 (47.4%)	15 (19.2%)	66	20 (30.3%)	29 (43.9)	7 (10.6)

**Table 10:** Effect of source of water on the prevalence of schistosomiasis in Abuja.

Source of Water	No. Examined	No. Positive (%)	*p* Value
Well/Rain	287	0 (0%)	<0.0001
Borehole	300	0 (0%)	
Tap water	238	0 (0%)	
River	324	203 (62.7%)	
Well/Rain/River	93	66 (71%)	
Well/Rain/borehole/Rivers	78	63 (80.8%)	
Borehole and Rivers	50	45 (90%)	
Total	1370	377 (27.5%)	

**Table 11:** Association between activities in the river and prevalence of schistosomiasis.

Activities in the River	Total Number Examined	No. Infected (%)	*p*-Value
Fetching	46	31 (67.4%)	<0.0001
Swimming	159	132 (83%)	
Bathing	94	58(61.7%)	
Washing	141	90 (63.8%)	
Crossing water	83	46 (55.4%)	
Fishing	22	20(90.9%)	
Total	545	377(69.2%)	

## Data Availability

The original contributions presented in this study are included in the article. Further inquiries can be directed to the corresponding authors.
